# Changes over time in population level transport satisfaction and mode of travel: A 13 year repeat cross-sectional study, UK

**DOI:** 10.1016/j.jth.2017.03.012

**Published:** 2017-09

**Authors:** Jonathan R. Olsen, Laura Macdonald, Anne Ellaway

**Affiliations:** a MRC/CSO Social and Public Health Sciences Unit, University of Glasgow, 200 Renfield Street, Glasgow G2 3QB, United Kingdom; b Centre for Research on Environment, Society and Health (CRESH), Institute of Health and Wellbeing, University of Glasgow, 1 Lilybank Gardens, Glasgow G12 8RZ, United Kingdom

**Keywords:** Transport, Travel satisfaction, Active travel, Urban areas, Environment, Health inequality

## Abstract

**Aim:**

The aim of the study was to examine changes over time in satisfaction with usual transport mode, explore individual and area level characteristics as mediators in the likelihood of transport satisfaction, and whether any changes in transport satisfaction varied by these factors over time.

**Methods:**

Adults from West Central Scotland, United Kingdom, who participated at both waves of the repeat cross-sectional ‘Transport, Health and Well-being Study’ conducted in 1997 (n=2735) and 2010 (n=2024) were assessed. Individuals completed a detailed postal questionnaire at both time points including self-rated satisfaction with usual transport mode (using a seven point scale subsequently dichotomised to a binary outcome of satisfied (1–2) and other (3–7)). Participants reported usual transport mode for travel to various destinations. A multilevel logistic regression model was used and individuals were nested within areas (c. 4000 population).

**Results:**

At the 2010 sweep, two thirds (n=1345) of individuals were satisfied with their transport choice. Those with fair/poor health were less satisfied with their usual transport compared to those in better health (Odds Ratio (OR) 0.49, p<0.001). Access to a car was associated with overall transport satisfaction (OR 2.63, p<0.001) and the effect of deprivation on transport satisfaction was mitigated when adjusted by household car access. Transport satisfaction increased more from 1997 to 2010 for retired individuals compared to those in employment (OR 1.40, p=0.032), and for those who travelled by public transport (OR 2.39, p=0.005) and using multiple modes (OR 2.19, p<0.001) compared to those who travelled by car.

**Conclusions:**

The proportion of those who travelled using public transport, active modes or by multiple mode increased journey satisfaction over time at a greater rate than those who travelled by car, highlighting that continued efforts should be made to promote these more active transport modes which have potential to impact on health.

## Highlights

• This study described change over time in satisfaction with usual transport mode from 1997 to 2010.
• Two third of individuals were satisfied with their usual transport for journeys.
• Those with poor or fair health experienced worse transport satisfaction.
• Access to a car at household increased transport satisfaction regardless of main mode.
• Continued efforts should be made to promote health beneficial active transport modes.

## 1 Introduction

Transport patterns, in terms of both access to and choice of transport, are structured by social inequality (Rydin et al., 2012), and lack of transport choice can lead to individuals (particularly those on low income) experiencing poor access to goods, services, and the inability to participate in society (Bocarejo and Oviedo, 2012). Knowledge of individuals’ use and experience of transport is important for furthering our understanding of the factors which are important for public health. Current public health strategies are aimed at encouraging the population to be more active (World Health Organisation, 2015), and active travel (walking, cycling) are viewed as having potential to reduce levels of obesity (Flint et al., 2016) and better mental health compared to travelling by car (Rissel et al., 2014). However, there is little known of the extent to which satisfaction with transport mode varies for different groups (e.g. gender, age, socio-economic status, employment, area of residence) and how this changes over time. Understanding transport satisfaction can inform national and local policy aimed at providing a sustainable transport infrastructure which can have important national and international benefits for both health and the environment (Cohen et al., 2014, Rydin et al., 2012, Sallis et al., 2016).

Travel mode choice is often dependent on where people live, for example those residing within suburban areas may be more likely to live in areas that they can travel to by car (De Vos et al., 2016). Whereas mixed-use neighbourhoods (i.e. mix of residential, service, commercial etc. uses) have facilities nearby that are easily accessed on foot (De Vos et al., 2016). The availability of transport varies between different community groups, which has implications for health (Mackett, 2014). Individual transportation decisions can also affect health and these decisions are not equal across neighbourhoods (American Public Health Association, 2016), those who experience the least benefit and most disbenefit from transport are often those already socially disadvantaged in many other ways (Cohen et al., 2014). This disadvantage has a historical context where those living in inner city areas that are generally serviced with good public transport infrastructure have suffered by the predominant development of streets for cars, meaning the reduction in safe routes for walking and cycling; this impact has been greatest for the poorest communities who traditionally relied on these free means of transport (Power, 2012). Outside of large urban centres, suburban low cost housing was often hastily constructed with inadequate public transport infrastructure resulting in those without access to a car having poor mobility (Power, 2012).

People may choose to live in an area that suits a lifestyle in terms of travel mode, access to various destinations, and dietary and physical activity preferences (Clark et al., 2010). However, a substantial proportion of people are unable to exercise choice (e.g. public sector housing tenants who are bound by the availability and location of public sector housing) and do not reside in their preferred neighbourhoods, and can therefore face difficulties in travelling by their preferred transport mode, particularly for utilitarian journeys rather than for leisure (Bergstad et al., 2011). Living in a rural or urban area can impact on health and wellbeing (Levasseur et al., 2015), through factors such as differences in transport provision and this can contribute to inequalities that influence health (Mackett, 2014). The built environment (both in urban and suburban neighbourhoods) can impose restrictions on the travel mode choice, often forcing disempowered residents to use a travel mode which is not the preferred one and sometimes older adults will move from rural to urban areas to have better access to services (Levasseur et al., 2015).

People may try to maximise their happiness and satisfaction when making transport mode choice, incorporating remembered experiences from previous decisions when contemplating future decisions (De Vos et al., 2016). Trip satisfaction is affected by both the characteristics of the trip and the individual (St-Louis et al., 2014), and the provision of good public transport is associated with higher levels of use (Dalton et al., 2013). However, it remains to be determined how close one's perception reflects reality, how the modes may differentially affect activity, and how to improve the understanding of the differences (Ding and Gebel, 2012).

It has been suggested that research should better conceptualise walking as an ambient activity; i.e. something enjoyed rather than incidental to everyday life (Whybrow, 2014) and that to encourage walking urban spaces should be constructed so that walking is both achievable and enjoyable (Pooley et al., 2014). For older people socio-economic factors are associated with utility cycling (journeys to work), however there is limited evidence of the role of socio-economic position with active travel (Heesch et al., 2014). The prevalence of active travel can increase for those without a car compared to those who have car access (Pucher et al., 2011); however there have been few studies that have adequately controlled for car ownership when describing transport mode choice. Many studies have used area based summaries of car ownership from national surveys rather than individual level data, particularly for active travel (Panter and Jones, 2010).

In building transport infrastructure which encourages journeys to be completed actively or using public transport infrastructure it is important to firstly understand current satisfaction with transport modes to a range of destinations, which adequately controls for car access, urbanicity, socio-economic and health status, and explore variations in these over time.

The aim of this study was to:

(1) Examine self-rated satisfaction with transport mode,
(2) Explore the impact of journey destination, sex, and age, education, car access, urban/rural classification, health and socio-economic status as moderators of the likelihood of transport satisfaction, and;
(3) Explore whether any changes in transport satisfaction over time varied by these factors.

## 2 Methodology

### 2.1 Study population

The data used were from our 2010 repeat of our 1997 postal survey ‘Transport, Housingand Well-being’ (THAW) of a random stratified sample of adults in eight local authority areas in the West of Scotland. THAW 2010 was based on THAW 1997, a study designed to examine three objectives, firstly, the statistical associations between long term morbidity and mental health and well-being on the one hand, and housing tenure and car ownership on the other (while controlling for socio-demographic and psychological characteristics); secondly, the role of housing quality, residential environment, and use of cars, in influencing illness and psychological health; and thirdly the meaning of housing tenure and car ownership in people's daily lives (Macintyre et al., 2003). THAW 2010 draws on respondents from the same geographical areas (due to its socially heterogeneous composition) to our 1997 postal survey and uses a very similar postal questionnaire to the previous study. As with our 1997 survey, our random sample of the general population was stratified to reduce selection bias (Sedgwick, 2015) using a geodemographic classification of neighbourhood type (using ACORN, Scottish version (CACI, 2010)) to ensure that all types of residential neighbourhoods (ranging from ‘affluent consumers in large houses’ to ‘poorest council estates’) were included in correct proportions.

The postal questionnaire (see online supplementary file), with three reminders (using Dillman's total design method (Dillman, 1978)) was sent out in the autumn of 2010. We achieved a response rate of 38% (2092 completed questionnaires, of whom 68 were excluded from the present analyses due to missing postcode data), from a sample of 5521 adults drawn from the electoral roll in the eight local authority areas which make up the Glasgow and Clyde Valley Structure Plan area in the West of Scotland. The estimated population in this area in 2010 was 1,763,430, and contains marked variations in social status and in health. (Audit Scotland, 2012) Survey respondents’ ages ranged from 17 to 95 years old. The socio-demographic characteristics of THAW 2010 were comparable to the previous THAW 1997 Study; e.g. respondents’ own social class was similar in THAW 1997 and THAW 2010 (65% and 70% in the non-manual social class groups, respectively). Compared to the West Central Scotland population, our achieved study sample characteristics were broadly similar for sex and for age; 56% were female, and 65% were of working age (18–60 years old), compared to 52% and 62% respectively within West Central Scotland (General Registrar Office, 2010). Within our sample, 85% of respondents had access to at least one car or van, while within the 2010 Scottish Household Survey, within West Central Scotland, 70% had access to a car (does not include van access) (Scottish Goverment, 2015).

THAW 2010 was approved by the Ethics Committee of the Faculty of Law, Business and Social Sciences at the University of Glasgow.

#### 2.1.1 Outcome variable – satisfaction with transport mode

Satisfaction with transport mode was measured using the Faces Scale (Andrews and Withey, 1976), a seven point scale with one being happiest and seven being unhappiest, and respondents were asked to tick the face which shows best how they feel about the means of transport they normally used to get around. The variable was transformed to create a binary outcome measure of ‘satisfied’ which included the two most positive outcomes (1 and 2) compared to ‘other’ (faces 3–7). The transformation allowed a robust and interpretable logistic regression model to be used for the analysis, and by using a binary outcome variable provided greater power than a 7-scale categorical outcome.

#### 2.1.2 Independent variables

The THAW survey collected a number of individual level variables such as sex, age (in years), self-rated health status over the past 12 months, and household car access. Age (in years) was grouped into the following four categories for analysis, 17 to 24, 25 to 44, 45 to 64 and 65+, and health status was dichotomised into ‘excellent/good’ or ‘fair/poor’. Each participant was linked to the six category Scottish urban/rural index (Scottish Government, 2014), and 2012 Scottish Index of Multiple Deprivation (SIMD) quintile (1=most deprived, 5=least deprived) (Scottish Government, 2012) based on the home postcode. The SIMD combines 38 indicators across the 7 domains, such as: income, employment, health, education, skills and training, housing, geographic access and crime (Scottish Government, 2012). The SIMD was also linked to individuals from the earlier 1997 THAW 1 cohort, and although this described area level deprivation for 2012, the SIMD index did not exist for the earlier wave. The alternative Carstairs Index, describing material disadvantage in the population using 2001 census data (Information services division (IDS) Scotland, 2010), was considered but ultimately not used due to two of the four main indicators being car ownership and unemployment, which were outcome variables in our analysis.

#### 2.1.3 Journey mode and destination

Participants provided information on their usual transport mode when travelling to the following destinations or events: day out, evening out, health appointment, sports facility, visiting friends/family, taking children to school, supermarket, and work/education. The journey destinations used in the THAW survey are similar to those used in other national travel surveys such as the UK National Travel Survey (Scottish Goverment, 2014).The modes for these journeys were categorised for analysis as: by car, public transport, active (walked or cycled) and multiple modes (a mixture of all modes). An overarching usual journey mode category was created based on the transport used for all journeys; if multiple modes were used across destinations the overall transport mode was selected. This was based on a description of UK daily travel in the 2010 National Travel Survey which showed that most journey purposes were for shopping, commuting and education (when commuting and education are combined, as in the THAW questionnaire) (Metz, 2010). Models were repeated individually for each destination and are presented in the paper to test this overarching travel assumption and explore differences between the overall and individual destination categories (Models 7–14, described in subsequent subsection).

### 2.2 Statistical analysis

A two-level logistic regression model was fitted where individuals were nested within Scottish intermediate geographies, which are geographical polygon areas based on home address of each participant (groups of approximately 4000 household residents which respect physical boundaries and natural communities, have a regular shape and contain households with similar social characteristics (Scottish Government, 2011)). Participants were matched to an intermediate geography area based on their home postcode being located within its boundary. This resulted in 402 intermediate geography areas for THAW 1 and 383 for THAW 2. An alternative smaller administrative boundary, Scottish data zone (administrative boundary of between 500–1000 households), was considered for the area level variable, however as 46.5% of data zones in THAW 2 (n=426/916) contained only 1 participant this removed the higher level context and thus was not chosen.

The dependent (outcome variable) was transport satisfaction (binary yes/no) and the independent covariates were travel mode, age, sex, health status, access to car, urban/rural classification, and socio-economic status.

All models specified individuals nested within intermediate geographies as a random effect. Statistical analyses were completed using MLwiN v2.36. The following models were performed for both survey waves and are presented in Table 2, Table 3:

*Model 1*: Null model.
*Model 2*: The following individual level variables were included as covariates: sex, age, and health status.
*Model 3*: Identical structure to ***model 2*** with contextual variables urban/rural classification and socio-economic status included.
*Model 4*: Identical structure to ***model 3*** with the variable access to car at household included.
*Model 5*: Identical structure to ***model 3*** with the variable journey mode (summary of all destinations) included.
*Model 6*: Identical structure to ***model 3*** with both the variable journey mode (summary of all destinations) and access to car at household included.
*Model 7–14*: To explore whether transport satisfaction differed by journey destination (rather than a summary of all destinations) and to test the assumptions of the overall transport mode category, an identical structure to ***model 6*** was repeated but the variable journey mode included separately for each destination: day out (model 7), evening out (model 8), attending health appointment (model 9), sports facility (model 10), visiting friends/family (model 11), taking children to school (model 12), supermarket (model 13), and work/education (model 14).

#### 2.2.1 Formal assessment of change in transport satisfaction over time

Size and significance of differences in change over time in reported transport satisfaction were assessed by interaction terms. Predictive margins of transport satisfaction were described and differences between the predictors of transport satisfaction reported.

## 3 Results

### 3.1 Participant characteristics

THAW 1 comprised a total of 2735 participants included in the analysis, of those 59% (n=1605) were female (Table 1), while for THAW 2 there was a smaller sample (n=2024) and a similar proportion were female (57%, n=1146)). The majority of participants were aged 25 to 44 in THAW 1 (36.1%, n=986) and 45 to 64 in THAW 2 (45%, n=912), and approximately a quarter were aged 65 and over at both surveys (THAW 1 27% (n=729), THAW 2: 25% (n=508)). A greater proportion of the sample rated their health as ‘excellent’ or ‘good’ in THAW 2 (70% (n=1400)) than in THAW 1 (60% (n=1, 615). Over half of the participants (THAW 1: 67% (n=1818), THAW 2 60% (n=1204)) lived in a large urban area, and around a quarter in both samples in other urban areas, representing the predominantly urban geography of West Central Scotland. There were slightly more participants living the least deprived quintile (25%, n=508) than the most deprived (20%, n=401). Most individuals (82%, n=1649) had access to a car at the household for the later time period.

**Table 1:** Participant characteristics and transport satisfaction.

**Variable**	**Wave 1 (1997)**		**Wave 2 (2010)**	
	**Participant characteristics**	**Transport satisfaction (N & % of total)**	**Participant characteristics**	**Transport satisfaction (N & % of total)**
	**(N & % of total)**		**(N & % of total)**	
Satisfied	2735 (100)	1531 (55.9)	2024 (100)	1345 (66.5)
***Sex***				
male	1130 (41.3)	644 (57.0)	878 (43.4)	594 (67.7)
female	1605 (58.7)	887 (55.3)	1146 (56.6)	751 (65.5)
***Age***				
17 to 24	116 (4.2)	60 (51.7)	109 (5.4)	58 (53.2)
25 to 44	986 (36.1)	598 (60.7)	487 (24.1)	314 (64.5)
45 to 64	901 (32.9)	526 (58.4)	912 (45.1)	627 (68.8)
65 plus	729 (26.7)	344 (47.2)	508 (25.1)	340 (66.9)
***Health status***				
Excellent/good	1615 (59.1)	1053 (65.2)	1400 (69.2)	1006 (71.9)
Fair/poor	1104 (40.4)	472 (42.8)	608 (30.0)	325 (53.5)
***Employment status***				
Employed or student	1282 (46.9)	829 (64.7)	1118 (55.2)	768 (68.7)
Unemployed	519 (19.0)	238 (45.9)	237 (11.7)	125 (52.7)
Retired	815 (29.8)	408 (50.1)	596 (29.4)	407 (68.3)
***Urban Rural classification***				
Large Urban Area	1818 (66.5)	976 (53.7)	1204 (59.5)	802 (66.6)
Other Urban Areas	667 (24.4)	414 (62.1)	539 (26.6)	359 (66.6)
Accessible Small Towns	160 (5.9)	93 (58.1)	175 (8.7)	116 (66.3)
Accessible Rural	82 (3.0)	44 (53.7)	92 (4.6)	55 (59.8)
Remote Rural	8 (0.3)	4 (50.0)	14 (0.7)	13 (92.9)
***SIMD Quintile***				
1 (Most Deprived)	909 (33.2)	433 (47.6)	401 (19.8)	228 (56.9)
2	627 (22.9)	338 (53.9)	428 (21.2)	271 (63.3)
3	435 (15.9)	262 (60.2)	388 (19.2)	258 (66.5)
4	324 (11.9)	200 (61.7)	299 (14.8)	213 (71.2)
5 (Least Deprived)	440 (16.1)	298 (67.7)	508 (25.1)	375 (73.8)
***Access to car at household***				
No access	959 (35.1)	*379 (39.5)*	364 (17.9)	169 (46.4)
Access	1640 (59.9)	1130 (68.9)	1649 (81.5)	1173 (71.1)
***Journey mode (All – summary)***				
Car	1118 (40.9)	*804 (71.9)*	1199 (59.2)	787 (65.6)
Public Transport	411 (15.0)	*133 (32.4)*	109 (5.4)	70 (64.2)
Active	367 (13.4)	*158 (43.1)*	65 (3.2)	42 (64.6)
Multiple	741 (27.1)	393 (53.0)	612 (30.2)	418 (68.3)

### 3.2 Transport satisfaction

Transport satisfaction was higher in the latter 2010 time period where two thirds (67%, n=1345) of all individuals were satisfied with their usual transport mode choice than during 1997 (56%, n=1531) (Table 1). This was slightly higher for males (THAW 1 57% (n=644), THAW 2 68% (n=594)) than females (THAW 1 55% (n=887), THAW 2 66% (n=751)) in both time periods. During the earlier time period, both the younger 17 to 24 (52%, n=60) and 65 plus (47%, n=344) age groups had the lowest proportion of transport satisfaction than the mid-life age groups. In the later 2010 time period transport satisfaction for the 64 plus age group consisted of the greatest proportion who were satisfied with their transport (70%, n=340) which was similar to the 25 to 44 (65%, n=314) and 45 to 64 (69%, n=627) age groups. The younger 17 to 24 age group had similarly lower reported levels of transport satisfaction in the 2010 survey (53%, n=58) as that reported in 1997 (52%, n=60).

Those who reported their health as excellent or good rated higher transport satisfaction (72%, n=1006) than those with fair or poor health (54%, n=325) in the 2010 survey. Similarly, those with access to a car at their household had a higher transport satisfaction (71%, n=1173) than those with no access (46%, n=169). Transport satisfaction was greater in less deprived areas.

There was little difference between journey mode and transport satisfaction when reported as a proportion for the most recent time period. The likelihood of transport satisfaction and rate of change over time between journey modes are described in the subsequent subsections.

### 3.3 Likelihood of transport mode satisfaction

Those who reported poor or fair health (THAW 1, Odds Ratio (OR) 0.51, p<0.001, THAW 2. OR 0.50, p<0.001) were less satisfied with their usual transport in all models and both time periods (Model 6, Tables 2 and 3). Household car access increased transport satisfaction (OR 2.63, p<0.001). For those unemployed in the latter 2010 time period having access to a car at the household removed the likelihood of dissatisfaction with transport (Table 2, Model 6). The effect of deprivation on transport satisfaction was mitigated when adjusted for household car access. There were no significant differences between gender, a combination variable of journey destination and transport satisfaction. In the earlier 1997 time period there were no differences in transport satisfaction and age, however in the latter 2010 time period individuals aged 25 and over were more likely to be satisfied with transport than the younger 17–24 age group (Table 3).

**Table 2:** Likelihood of journey satifaction, individual level variables, contextual level variables, and summary of journey destination THAW 1 (1997).

**Likelihood of transport satisfaction (Wave 1)**			**Model 1**		**Model 2**		**Model 3**		**Model 4**		**Model 5**		**Model 6**	
			**Null model**		**Individual variables**		**Model 2+Contextual variables**		**Model 3+Car Access**		**Model 3+Usual journey mode**		**Model+Car Access & Usual journey mode**	
**Variable**	**N (%)**		**OR (LL – UL 95% CI)**	**p**	**OR (LL – UL 95% CI)**	**p**	**OR (LL – UL 95% CI)**	**p**	**OR (LL – UL 95% CI)**	**p**	**OR (LL – UL 95% CI)**	**p**	**OR (LL – UL 95% CI)**	**p**
***Sex***														
Male	1130 (41.3)	***Ref***												
Female	1605 (58.7)				0.99 (0.84 to 1.16)	0.909	0.99 (0.84 to 1.16)	0.860	1.07 (0.90 to 1.26)	0.443	1.06 (0.89 to 1.25)	0.528	1.09 (0.92 to 1.30)	0.297
***Age***														
17–24	116 (4.2)	***Ref***												
25–44	986 (36.1)				***1.53 (1.02 to 2.28)***	***0.038***	1.48 (1.00 to 2.21)	0.053	1.46 (0.97 to 2.20)	0.072	1.30 (0.87 to 1.96)	0.205	1.33 (0.88 to 2.00)	0.181
45–64	901 (32.9)				***1.75 (1.16 to 2.64)***	***0.008***	***1.70 (1.12 to 2.56)***	***0.012***	***1.76 (1.15 to 2.69)***	***0.009***	1.43 (0.94 to 2.19)	0.095	1.51 (0.99 to 2.32)	0.058
65 plus	729 (26.7)	3 missing			1.18 (0.72 to 1.93)	0.518	1.14 (0.70 to 1.88)	0.598	1.51 (0.90 to 2.53)	0.116	1.01 (0.61 to 1.69)	0.958	1.20 (0.71 to 2.03)	0.488
***Health status***														
Excellent/good	1615 (59.1)	***Ref***												
Fair/poor	1104 (40.4)	16 missing			***0.45 (0.39 to 0.54)***	***<0.001***	***0.48 (0.40 to 0.57)***	***<0.001***	***0.52 (0.44 to 0.63)***	***<0.001***	***0.48 (0.40 to 0.57)***	***<0.001***	***0.51 (0.43 to 0.62)***	***<0.001***
***Employment status***														
Employed or student	1282 (46.9)	***Ref***												
Unemployed	519 (19.0)				***0.63 (0.50 to 0.79)***	***<0.001***	***0.70 (0.55 to 0.88)***	***0.002***	0.83 (0.65 to 1.06)	0.140	***0.76 (0.59 to 0.97)***	***0.028***	***0.77 (0.60 to 1.00)***	***0.048***
Retired	815 (29.8)	119 missing			0.82 (0.59 to 1.13)	0.220	0.86 (0.62 to 1.18)	0.344	0.98 (0.70 to 1.38)	0.926	0.99 (0.71 to 1.39)	0.963	0.99 (0.70 to 1.40)	0.966
***Urban Rural classification***														
Large urban area	1818 (66.5)	***Ref***												
Other urban areas	667 (24.4)						***1.29 (1.05 to 1.58)***	***0.014***	1.22 (1.00 to 1.50)	0.054	***1.27 (1.03 to 1.56)***	***0.022***	***1.23 (1.00 to 1.51)***	***0.047***
Accessible small towns	160 (5.9)						1.01 (0.71 to 1.44)	0.951	0.98 (0.68 to 1.40)	0.901	1.00 (0.70 to 1.44)	0.993	1.00 (0.70 to 1.44)	0.997
Accessible rural	82 (3.0)						0.93 (0.58 to 1.51)	0.780	0.71 (0.44 to 1.14)	0.156	0.79 (0.49 to 1.29)	0.352	0.69 (0.42 to 1.12)	0.133
Remote rural	8 (0.3)						0.44 (0.10 to 1.90)	0.271	0.36 (0.09 to 1.52)	0.166	0.58 (0.14 to 2.46)	0.457	0.51 (0.12 to 2.11)	0.349
***SIMD quintile***														
1 (Most deprived)	909 (33.2)	***Ref***												
2	627 (22.9)						1.12 (0.90 to 1.40)	0.309	0.92 (0.73 to 1.16)	0.491	0.97 (0.77 to 1.22)	0.779	0.89 (0.71 to 1.12)	0.331
3	435 (15.9)						***1.33 (1.03 to 1.72)***	***0.027***	1.07 (0.82 to 1.39)	0.614	1.10 (0.84 to 1.42)	0.496	1.01 (0.78 to 1.32)	0.923
4	324 (11.9)						1.30 (0.98 to 1.72)	0.067	0.87 (0.65 to 1.17)	0.362	0.93 (0.69 to 1.24)	0.623	0.80 (0.60 to 1.08)	0.146
5 (Least deprived)	440 (16.1)	6 missing					***1.68 (1.30 to 2.19)***	***<0.001***	1.00 (0.76 to 1.32)	0.984	1.10 (0.84 to 1.45)	0.491	0.91 (0.69 to 1.20)	0.502
***Access to car at household***														
No access	959 (35.1)	***Ref***												
Access	1640 (59.9)	136 missing							***2.97 (2.44 to 3.62)***	***<0.001***			***1.77 (1.38 to 2.27)***	***<0.001***
***Journey mode (journey destination summary)***														
Car	1118 (40.9)	***Ref***												
Public transport	411 (15.0)										***0.24 (0.18 to 0.31)***	***<0.001***	***0.34 (0.25 to 0.47)***	***<0.001***
Active	367 (13.4)										***0.34 (0.26 to 0.45)***	***<0.001***	***0.49 (0.36 to 0.67)***	***<0.001***
Multiple	741 (27.1)	98 missing									***0.41 (0.33 to 0.50)***	***<0.001***	***0.46 (0.37 to 0.58)***	***<0.001***
ICC			0.044		0.025		0.016		0.007		0.011		0.005	
R-squared			<0.0001		0.075		0.089		0.171		0.158		0.201	
MOR			1.45		1.32		1.25		1.16		1.21		1.32	

*Note*: Binomial distribution, 2nd order linearisation and PQL estimation. OR: Odds ratio, LL: lower level, UL: upper level, CI: confidence level.

**Table 3:** Likelihood of journey satifaction, individual level variables, contextual level variables, and summary of journey destination THAW 2 (2010).

**Likelihood of transport satisfaction (Wave 2)**			**Model 1**		**Model 2**		**Model 3**		**Model 4**		**Model 5**		**Model 6**	
			**Null model**		**Individual variables**		**Model 2+Contextual variables**		**Model 3+Car Access**		**Model 3+Usual journey mode**		**Model+Car Access & Usual journey mode**	
**Variable**	**N (%)**		**OR (LL – UL 95% CI)**	**p**	**OR (LL – UL 95% CI)**	**p**	**OR (LL – UL 95% CI)**	**p**	**OR (LL – UL 95% CI)**	**p**	**OR (LL – UL 95% CI)**	**p**	**OR (LL – UL 95% CI)**	**p**
***Sex***														
Male	878 (43.4)	***Ref***												
Female	1146 (56.6)				0.93 (0.76 to 1.13)	0.398	0.92 (0.75 to 1.12)	0.459	0.96 (0.79 to 1.17)	0.692	0.91 (0.75 to 1.11)	0.354	0.95 (0.78 to 1.17)	0.634
***Age***														
17–24	109 (5.4)	***Ref***												
25–44	487 (24.1)				***1.66 (1.07 to 2.56)***	***0.019***	***1.70 (1.10 to 2.62)***	***0.017***	***1.61 (1.03 to 2.51)***	***0.035***	***1.71 (1.10 to 2.66)***	***0.016***	***1.63 (1.04 to 2.54)***	***0.031***
45–64	912 (45.1)				***2.18 (1.07 to 3.31)***	***<0.001***	***2.21 (1.45 to 3.38)***	***<0.001***	***2.14 (1.40 to 3.30)***	***<0.001***	***2.28 (1.49 to 3.48)***	***<0.001***	***2.19 (1.43 to 3.37)***	***<0.001***
65 plus	508 (25.1)	8 missing			***2.18 (1.43 to 3.25)***	***0.024***	***1.87 (1.08 to 3.25)***	***0.026***	***2.27 (1.27 to 3.92)***	***<0.001***	***1.91 (1.10 to 3.33)***	***0.022***	***2.26 (1.28 to 3.97)***	***0.005***
***Health status***														
Excellent/good	1400 (69.2)	***Ref***												
Fair/poor	608 (30.0)	16 missing			***0.45 (0.36 to 0.56)***	***<0.001***	***0.49 (0.39 to 0.61)***	***<0.001***	***0.51 (0.41 to 0.63)***	***<0.001***	***0.48 (0.39 to 0.60)***	***<0.001***	***0.50 (0.40 to 0.63)***	***<0.001***
***Employment status***														
Employed or student	1118 (55.2)	***Ref***												
Unemployed	237 (11.7)				***0.67 (0.49 to 0.91)***	***0.011***	***0.72 (0.52 to 0.98)***	***0.036***	0.91 (0.65 to 1.26)	0.573	***0.71 (0.52 to 0.97)***	***0.033***	0.90 (0.65 to 1.25)	0.539
Retired	596 (29.4)				1.10 (0.76 to 1.59)	0.616	1.10 (0.76 to 1.61)	0.605	1.13 (0.77 to 1.66)	0.528	1.11 (0.76 to 1.61)	0.594	1.14 (0.77 to 1.67)	0.515
***Urban Rural classification***														
Large urban area	1204 (59.5)	***Ref***												
Other urban areas	539 (26.6)						0.95 (0.74 to 1.21)	0.659	0.94 (0.73 to 1.21)	0.636	0.91 (0.70 to 1.18)	0.486	0.90 (0.69 to 1.18)	0.458
Accessible small towns	175 (8.7)						0.93 (0.63 to 1.37)	0.719	0.83 (0.56 to 1.24)	0.365	0.95 (0.64 to 1.42)	0.819	0.85 (0.56 to 1.27)	0.420
Accessible rural	92 (4.6)						0.68 (0.41 to 1.12)	0.129	***0.59 (0.36 to 0.98)***	***0.041***	0.68 (0.41 to 1.13)	0.134	***0.58 (0.35 to 0.98)***	***0.041***
Remote rural	14 (0.7)						6.08 (0.73 to 50.80)	0.096	5.27 (0.62 to 44.66)	0.127	6.49 (0.80 to 54.63)	0.086	5.54 (0.65 to 47.14)	0.117
***SIMD quintile***														
1 (Most deprived)	401 (19.8)	***Ref***												
2	428 (21.2)						1.30 (0.96 to 1.76)	0.090	1.09 (0.80 to 1.50)	0.576	1.28 (0.94 to 1.74)	0.111	1.09 (0.79 to 1.49)	0.603
3	388 (19.2)						***1.39 (1.00 to 1.92)***	***0.047***	1.16 (0.83 to 1.62)	0.394	1.36 (0.98 to 1.88)	0.066	1.14 (0.81 to 1.60)	0.444
4	299 (14.8)						***1.58 (1.12 to 2.24)***	***0.010***	1.28 (0.89 to 1.83)	0.186	***1.57 (1.11 to 2.23)***	***0.012***	1.27 (0.88 to 1.82)	0.197
5 (Least deprived)	508 (25.1)	6 missing					***1.81 (1.33 to 2.48)***	***<0.001***	1.34 (0.96 to 1.86)	0.086	***1.81 (1.32 to 2.48)***	***<0.001***	1.34 (0.96 to 1.87)	0.084
***Access to car at household***														
No access	364 (17.9)	***Ref***												
Access	1649 (81.5)	11 missing							***2.60 (1.98 to 3.40)***	***<0.001***			***2.57 (1.96 to 3.37)***	***<0.001***
***Journey mode (journey destination summary)***														
Car	1199 (59.2)	***Ref***												
Public transport	109 (5.4)										1.11 (0.69 to 1.79)	0.656	1.13 (0.70 to 1.84)	0.615
Active	65 (3.2)										1.18 (0.65 to 2.13)	0.582	1.07 (0.59 to 1.95)	0.824
Multiple	612 (30.2)	39 missing									1.24 (0.98 to 1.58)	0.074	1.20 (0.94 to 1.53)	0.138
ICC			0.029		0.027		0.027		0.031		0.028		0.032	
R-squared			<0.0001		0.048		0.063		0.088		0.067		0.091	
MOR			2.75		2.74		2.74		2.76		2.75		2.77	

*Note*: Binomial distribution, 2nd order linearisation and PQL estimation. OR: Odds ratio, LL: lower level, UL: upper level, CI: confidence level.

Supplementary Table 2 contains models 7–14 which describe the likelihood of transport satisfaction for individual journey destinations to explore whether transport satisfaction differed by transport mode during 2010 (THAW 1). There were no substantial changes in the main results for both the individual and area level variables. There were changes in the likelihood of transport satisfaction, compared to travelling by car, for journeys travelled using mixed mode for a day out (OR: 1.46, p=0.011), visiting family/friends (OR: 1.50, p=0.005), and to work/education (OR: 1.59, p=0.038). Journeys using public transport for travel to work/education resulted in improved transport satisfaction (OR: 1.54, p=0.017). Supplementary Table 1 presents transport satisfaction for individual journey destinations reported in the 1997 sweep.

### 3.4 Changes in the likelihood of transport satisfaction over time (1997–2010)

Table 4 compares change over time from 1997 to 2010 in the likelihood of transport satisfaction. The ORs are from interaction terms and provide a means of formally assessing whether the change over time in each category is significantly different to that in the reference categories. For example, the OR for females indicates whether the increase in likelihood of transport satisfaction over time for women is significantly different to the increase over time for men. The table shows that there were no significant differences between genders, age groups, or health status in change over time. However, the likelihood of transport satisfaction increased by a greater amount for those retired than those in employment (OR 1.40, p=0.032), for living in remote rural areas compared to large urban areas (OR 10.54, p=0.007), and journeys travelled by public transport (OR 2.39, p=0.005) and using multiple modes (OR 2.19, p<0.001) compared to journeys by car. Active travel showed borderline significance in increases in transport mode satisfaction compare to car (OR 1.86, p=0.054).

**Table 4:** Difference in change in the likelihood of transport satisfaction over time (1997–2010) relative to the size of the difference in the base category.

	**Interaction over time**			
	**OR**	**p**	**LL 95% CI**	**UL 95% CI**
***Gender***				
Male	**Ref**			
Female	0.90	0.440	0.70	1.17
***Age band***				
17–24	**Ref**			
25–44	1.12	0.718	0.61	2.03
45–64	1.25	0.450	0.70	2.25
65 plus	1.69	0.093	0.92	3.14
***Health status***				
Excellent/good	**Ref**			
Fair/poor	1.00	1.000	0.77	1.30
***Employment status***				
Employed or student	**Ref**			
Unemployed	1.08	0.682	0.75	1.54
Retired	**1.40**	**0.032**	**1.03**	**1.9**
***Urban Rural classification***				
Large urban area	**Ref**			
Other urban areas	0.85	0.330	0.61	1.18
Accessible small towns	0.87	0.643	0.5	1.54
Accessible rural	0.92	0.881	0.44	1.89
Remote rural	**10.54**	**0.007**	**1.93**	**57.7**
***SIMD quintile***				
1 (Most deprived)	**Ref**			
2	1.16	0.447	0.79	1.71
3	1.06	0.770	0.73	1.52
4	**1.53**	**0.050**	**1.00**	**2.34**
5 (Least deprived)	1.34	0.176	0.88	2.04
***Access to car at household***				
No access	**Ref**			
Access	1.08	0.662	0.77	1.51
***Journey mode***				
Car	**Ref**			
Public transport	**2.39**	**0.005**	**1.30**	**4.41**
Active	1.86	0.053	0.99	3.49
Multiple	**2.19**	**<0.001**	**1.57**	**3.01**

## 4 Discussion

### 4.1 Main findings

The aim of our study was to examine transport satisfaction with usual mode of transport, explore the impact of journey destination, sex, age, car access, urban/rural classification, health and socio-economic status as moderators of the likelihood of transport satisfaction, and explore any changes in transport satisfaction in these variables over time. We found that in 2010, two thirds of individuals were satisfied with their transport mode choice. The main moderators in the likelihood of increased transport satisfaction in 2010 were older age, good or excellent health, and household car access. The effect of area level deprivation on the likelihood of transport satisfaction was mitigated when adjusted for access to car at household.

Transport satisfaction increased from 1997 to 2010 for those retired and the older 65+ age group who, interestingly, reported the least satisfaction at the 1997 survey. This improvement in transport satisfaction over time remained significant only for those retired compared to those employed or in full time education.

There were greater increases in transport satisfaction for journeys completed by public transport, using multiple modes and actively travelled when compared to journeys made by car.

### 4.2 Comparison with existing literature

There are many interventions that are required to create healthier and more sustainable cities for the future, two of which are improving access to public transport, and enhancing the desirability of active travel (Giles-Corti et al., 2016). Our study of transport satisfaction in the West of Scotland found that transport satisfaction had increased over time and that these increases were greatest when travelling by public transport, active transport and using multiple transport modes, compared to car transport. Other recent studies have shown that it is often subjective attitudes which influence commuter travel satisfaction, and those who feel travel has a purpose rather than a ‘waste of time’ are often more satisfied with travel (Ye and Titheridge, 2016). Environmentally friendly commuters are often the most satisfied with their travel and many studies have shown that cycling and walking commuters have the greatest levels transport satisfaction (Rissel et al., 2015, Smith, 2016, Ye and Titheridge, 2016). Although our study did not show active travel having the greatest reported transport satisfaction overall, the proportion reporting satisfaction in transport when using this mode did increase over time compared to satisfaction with car transport. Our study also included all journeys for multiple purposes where many studies have described transport satisfaction only for commuter travel, when we analysed journey satisfaction for which the main journey purpose was travel to work or education in the most recent time period we did not find increased nor decreased satisfaction for active travel compared to those whose main mode for that purpose was car.

We found an increase in journey satisfaction where multiple modes of transport were used compared to car travel alone. This suggests that individuals are potentially enjoying more flexible, multimode travel, including different journey modes when travelling to a destination. Dalton et al. (2013) recommend the use of existing public transport networks to break car journeys, such as ‘park and ride’ schemes or places which facilitate the change from active modes to public transport. Our findings supports the promotion of this multi-mode transport policy encouraging mixed transport modes, as these provided positive levels of transport satisfaction.

Our findings suggested that household car access was associated with greater transportation satisfaction overall, even where an alternative transport mode, such as public transport, displayed greater satisfaction for a specific journey destination. Qualitative studies in Montreal, Canada, showed that individuals wished to have access to a car for journeys when they feel it is required and transport satisfaction was improved when the journey decision was a personal choice (St-Louis et al., 2014). Therefore, an individual being empowered to choose whether or not to travel by car may be an indicator of overall greater transport satisfaction. For example, having the option to use a car for instances where it may be raining or the requirement of transporting lots of baggage which may be difficult when using other modes (Levasseur et al., 2015).

Alternatively, researchers have hypothesised that households unable to use a car could be more satisfied with their daily travel as they have adjusted their activity schedule so they have little need for travel where the car is superior, or they have chosen to live in neighbourhoods with satisfactory public transport services (Bergstad et al., 2011). Our finding differs from this hypothesis, and the deprivation indicator used in the analysis included a measure of geographical access, therefore adjusting for access to public transport services. Those without a car may feel disempowered in their transport decisions by being unable to participate in activities requiring travel by car, leading to lower transport satisfaction.

Studies have recommended understanding attitudes of older people towards transport (Levasseur et al., 2015) and we found that older adults were more satisfied with their usual transport mode than younger individuals (16–24 old) in the later 2010 survey. However, a key determinant of transport satisfaction was health, poor health is more prevalent in older age as life expectancy rises at a greater rate than health life expectancy (Rechel et al., 2013), meaning that the population are growing older but often not living these prolonged years in good health, particularly among those in deprived areas. Therefore this age group may be most susceptible for being dissatisfied with their usual transport.

The likelihood of transport satisfaction showed a greater increase from 1997 to 2010 for those retired compared to people in employment or full time education. From 1st April 2006 the Scottish Government introduced the *‘Scotland-wide Free Bus Travel Scheme for Older and Disabled People’* (Scottish Goverment, 2010), and this may have contributed to the increase in transport satisfaction for public transport and by individuals who are retired over this time period. Studies evaluating the impact of concessionary bus fares for older people showed that the policy produced modal shifts from car to bus, but the biggest increases in usage were amongst more affluent car-owning male pensioners (Rye and Mykura, 2009). The evaluation also showed limited evidence of increased social inclusion for those living in the most deprived areas (Rye and Mykura, 2009), and others have warned that those most isolated are unable to use the bus (Andrews et al., 2012). As well as applying caution to the overall positive effects of free bus travel to all, there are other potential positives for older people resulting from free bus travel policies, such as providing significant health benefits through increased incidental physical activity (Coronini-Cronberg et al., 2012).

We found higher transport satisfaction for journeys to work or education if the usual mode was public transport or a combination of multiple transport modes. Other studies have found that train commuters, as well as pedestrians and cyclists, were significantly more satisfied than car users in their journey mode (St-Louis et al., 2014). This may be due to public transport providing a more structured journey which the individual has tailored to suit their needs. It is also important to consider that car journeys during peak rush hour commuter times have a higher frequency of traffic jams which can cause additional stress and therefore decreased transport satisfaction (Wener and Evans, 2011) and roads in Scotland had experienced a substantial increase in traffic volume during the study period of 16% from 1996 to 2014 (Transport Scotland, 2014). Alternative travel by transport modes such as train show decreased levels of stress and less negative moods (Wener and Evans, 2011). Higher satisfaction for public transport to a work or education destination is a positive finding in encouraging sustainable transport options for commuters, and efforts should continue to improve and promote public transport. In addition to the increased congestion on roads, which may have resulted in decreased satisfaction for car drivers, between the 1997 (wave 1) and 2010 (wave 2) there had been investment in cycling infrastructure across the UK, and more specifically in the West of Scotland. In 2008 Sustrans, a UK wide cycling charity, and Transport Scotland, The Scottish Government, formally monitored a three-year £16 million investment in cycling infrastructure across Scotland (Sustrans Scotland, 2011). Cycling commuters are generally more satisfied with their travel than road users (De Vos et al., 2016), and improved cycling infrastructure in the region could have been an important factor in increased transport satisfaction.

Due to small numbers, we were unable to distinguish between walking and cycling commuters. Studies have shown that for those living in London walking commuters were more satisfied than those cycling (Chng et al., 2016). Outside of areas where commuting by bicycle have experienced large increases, such as London, for the full population of England and Wales the proportion of journeys to work on bicycle constitute a very small proportion of all commuter journeys (2.8%) and has remained the same from 2001 to 2011 (Office for National Statistics (ONS), 2014).

### 4.3 Strengths and limitations

The strength of our study was our ability to measure individual and contextual level characteristics which may influence transport satisfaction. We were also able to describe changes in transport satisfaction for populations living in the same areas, over time. The THAW study contains a representative sample from West Central Scotland and postcode data of each participant were collected. This allowed for area level variables, such as deprivation and urban/rural geography, to be matched to individuals. In addition to these data, each individual completed a questionnaire which recorded individual characteristics such as gender, age, health status, and access to car at household. Therefore allowing us to use precise individual measures rather than assigning an area level characteristic using sources such as the census. We included a self-reported measure of household car access; this has been a limitation of recent studies which have not included this variable in their statistical analysis. We were also able to distinguish between the various journey destinations individuals undertake in their day to day lives, whereas most previous studies have often described commuter journeys only.

The THAW respondents were representation of the West of Scotland, and other urban areas of Scotland, as described earlier in this paper (Section 2.1 Study population). Two thirds of participants were in the upper non-manual social classes, and as with all regional surveys limitations should be applied when applying these results to other developed regions. Other world-wide studies in Belgium (De Vos et al., 2016), Australia (Rissel et al., 2015), China (Ye and Titheridge, 2016), Canada (St-Louis et al., 2014) and the USA (Smith, 2016), have shown increased transport satisfaction when using active travel modes compared to those who drive, some also for public transport. However, understanding transport satisfaction is a complex task and is determined by multiple factors (St-Louis et al., 2014), including the origin and destination of a trip on a local, city and world-wide scale where transport infrastructure differs.

Our analysis used a two level logistic regression model; this allowed us to complete the analysis accounting for variance at an area level. An appropriate area level variable was chosen based on the number of individuals nested within.

The study does have limitations. Transportation satisfaction was rated in one overall ‘smiley face’ scale; this was subsequently dichotomised into a binary outcome of satisfied/other. The questions asked individuals to rate overall transport satisfaction for their usual transport mode and then provide detail of the usual transport mode used for a range of destinations. Therefore assumptions have been made in assigning transport satisfaction to each journey mode and destination; however the analysis was repeated in a range of models for individual destinations to explore differences between these and transport satisfaction.

A potential limitation of the THAW questionnaire when describing transport satisfaction was that the participants were asked to describe satisfaction for their ‘usual journey’, and this has both positives and negatives in its application. The survey question used generated a general response drawing on all journeys from previous days, months or years to describe how participants rated their overall transportation feelings. This may not be wholly accurate of their satisfaction due to the time lag between journeys and completing the questionnaire. An alternative question, such as describing satisfaction for their most recent trip, would have instructed participants to remember their transport satisfaction over a shorter a time period which may have produced a more accurate response. However, this most recent trip may have been an unusually bad experience, therefore not representative of their general feelings of usual transport mode.

The area-level deprivation variable (SIMD) included 38 variables across seven domains, which contained a measure of employment. This could lead to multicollinearity between the individual level predictor variable ‘employment status’. When performing models one to six we found no substantial changes in the estimated regression coefficients when predictor variables were added or deleted, to suggest any multicollinearity.

### 4.4 Conclusions

In conclusion, most individuals were satisfied with their journey transportation. Those with poor or fair health experienced worse transport satisfaction than those in good or excellent health, highlighting that further efforts are required to improve transport for those with worst health. Access to a car at the household improved transport satisfaction in all models, suggesting that empowerment to make a transport decision, even when other modes are more favourable to a car, improves overall transport satisfaction. However, when this was examined by type of journey, commuter journeys using public transport showed greater transport satisfaction than those by car in 2010 compared to 1997, highlighting continued efforts should be made to promote this transport mode.

Transport satisfaction improved from 1997 to 2010 for those retried and the older 65+ age group, who were the least satisfied during the first time period. Suggesting policies in Scotland, such as the free bus travel for older and disabled individuals, have improved transport satisfaction and has the potential to provide health benefits if adapted into international policy.

We found that increases in transport satisfaction were greater for journeys by public transport, by active or multiple modes, compared to those by car. Highlighting opportunities for promotion of these transport modes, and mixed mode transport, as a more satisfactory journey.

Future studies should enhance the understanding of transport satisfaction by including the length of journey and precise transport mode (bus/train/subway) for each journey.

## Funding statement

JO, LM and AE are employed by the University of Glasgow and funded as part of the Neighbourhoods and Communities Programme (MC_UU_12017/10) at the MRC/CSO Social and Public Health Sciences Unit (SPHSU).

## Authors’ contributions

JO led the design of the study with contributions from AE, and all authors contributed to the interpretation of analysis. LM conducted the data input and cleaning of the THAW dataset. JO conducted the analysis and first draft of the paper, with all authors contributing to its main content and revising it with critical comments. All authors have read and approved the manuscript prior to submission, and agree to be accountable for all aspects of the work in ensuring that questions related to the accuracy or integrity of any part of the work are appropriately investigated and resolved.

## Competing interests

The authors declare no conflicts.

## Data sharing statement

For further information please refer to the MRC/CSO Social and Public Health Sciences Unit data sharing statement at http://thaw.sphsu.mrc.ac.uk/information-for-researchers.

## References

[bib1] American Public Health Association (2016). Transportation and Health.

[bib2] Andrews F.M., Withey S.B. (1976). Social Indicators of Well-being: Americans' Perceptions of Life Quality.

[bib3] Andrews G., Parkhurst G., Susilo Y.O., Shaw J. (2012). The grey escape: investigating older people's use of the free bus pass. Transp. Plan. Technol..

[bib4] Audit Scotland (2012). Health Inequalities in Scotland.

[bib5] Bergstad C.J., Gamble A., Gärling T., Hagman O., Polk M., Ettema D., Friman M., Olsson L.E. (2011). Subjective well-being related to satisfaction with daily travel. Transportation.

[bib6] Bocarejo S.J.P., Oviedo H.D.R. (2012). Transport accessibility and social inequities: a tool for identification of mobility needs and evaluation of transport investments. J. Transp. Geogr..

[bib7] CACI, 2010. A Classification of Residential Neighbourhoods.

[bib8] Chng S., White M., Abraham C., Skippon S. (2016). Commuting and wellbeing in London: the roles of commute mode and local public transport connectivity. Prev. Med..

[bib9] Clark M.I., Berry T.R., Spence J.C., Nykiforuk C., Carlson M., Blanchard C. (2010). Key stakeholder perspectives on the development of walkable neighbourhoods. Health Place.

[bib10] Cohen J.M., Boniface S., Watkins S. (2014). Health implications of transport planning, development and operations. J. Transp. Health.

[bib11] Coronini-Cronberg S., Millett C., Laverty A.A., Webb E. (2012). The impact of a free older persons' bus pass on active travel and regular walking in England. Am. J. Public Health.

[bib12] Dalton A.M., Jones A.P., Panter J.R., Ogilvie D. (2013). Neighbourhood, route and workplace-related environmental characteristics predict adults' mode of travel to work. PLoS One.

[bib13] De Vos J., Mokhtarian P.L., Schwanen T., Van Acker V., Witlox F. (2016). Travel mode choice and travel satisfaction: bridging the gap between decision utility and experienced utility. Transportation.

[bib14] Dillman D. (1978). Mail and Telephone Surveys; the Total Design Method.

[bib15] Ding D., Gebel K. (2012). Built environment, physical activity, and obesity: what have we learned from reviewing the literature?. Health Place.

[bib16] Flint E., Webb E., Cummins S. (2016). Change in commute mode and body-mass index: prospective, longitudinal evidence from UK Biobank. Lancet Public Health.

[bib17] General Registrar Office, 2010. GROS Mid-2010 Population Estimates Scotland, Edinburgh.

[bib18] Giles-Corti B., Vernez-Moudon A., Reis R., Turrell G., Dannenberg A.L., Badland H., Foster S., Lowe M., Sallis J.F., Stevenson M. (2016). City planning and population health: a global challenge. Lancet.

[bib19] Heesch K.C., Giles-Corti B., Turrell G. (2014). Cycling for transport and recreation: associations with socio-economic position, environmental perceptions, and psychological disposition. Prev. Med..

[bib20] Information services division (IDS) Scotland (2010). The Carstairs and Morris Index.

[bib21] Levasseur M., Généreux M., Bruneau J.-F., Vanasse A., Chabot É., Beaulac C., Bédard M.-M. (2015). Importance of proximity to resources, social support, transportation and neighborhood security for mobility and social participation in older adults: results from a scoping study. BMC Public Health.

[bib22] Macintyre S., Ellaway A., Hiscock R., Kearns A., Der G., McKay L. (2003). What features of the home and the area might help to explain observed relationships between housing tenure and health? Evidence from the west of Scotland. Health Place.

[bib23] Mackett R.L. (2014). The health implications of inequalities in travel. J. Transp. Health.

[bib24] Metz D. (2010). Saturation of demand for daily travel. Transp. Rev..

[bib25] Office for National Statistics (ONS) (2014).

[bib26] Panter J.R., Jones A. (2010). Attitudes and the environment as determinants of active travel in adults: what do and don’t we know. J. Phys. Act. Health.

[bib27] Pooley C.G., Horton D., Scheldeman G., Mullen C., Jones T., Tight M. (2014). ‘You feel unusual walking’: the invisible presence of walking in four English cities. J. Transp. Health.

[bib28] Power A. (2012). Social inequality, disadvantaged neighbourhoods and transport deprivation: an assessment of the historical influence of housing policies. J. Transp. Geogr..

[bib29] Pucher J., Buehler R., Merom D., Bauman A. (2011). Walking and cycling in the United States, 2001–2009: evidence from the National Household Travel Surveys. Am. J. Public Health.

[bib30] Rechel B., Grundy E., Robine J.-M., Cylus J., Mackenbach J.P., Knai C., McKee M. (2013). Ageing in the European union. Lancet.

[bib31] Rissel C., Crane M., Wen L.M., Greaves S., Standen C. (2015). Satisfaction with transport and enjoyment of the commute by commuting mode in inner Sydney. Health Promot. J. Aust..

[bib32] Rissel C., Petrunoff N., Wen L., Crane M. (2014). Travel to work and self-reported stress: findings from a workplace survey in south west Sydney, Australia. J. Transp. Health.

[bib33] Rydin Y., Bleahu A., Davies M., Dávila J.D., Friel S., De Grandis G., Groce N., Hallal P.C., Hamilton I., Howden-Chapman P. (2012). Shaping cities for health: complexity and the planning of urban environments in the 21st century. Lancet.

[bib34] Rye T., Mykura W. (2009). Concessionary bus fares for older people in Scotland–are they achieving their objectives?. J. Transp. Geogr..

[bib35] Sallis J.F., Bull F., Burdett R., Frank L.D., Griffiths P., Giles-Corti B., Stevenson M. (2016). Use of science to guide city planning policy and practice: how to achieve healthy and sustainable future cities. Lancet.

[bib36] Scottish Goverment, 2010. Scotland-wide Free Bus Travel Scheme. Edinburgh, Scotland.

[bib37] Scottish Goverment, 2014. National Travel Survey: Scottish Results – All Editions. Scottish Government, Edinburgh, Scotland.

[bib38] Scottish Goverment, 2015. Scottish Household Survey.

[bib39] Scottish Government, 2011. Scottish Neighbourhood Statistics: Intermediate Geography Background Information.

[bib40] Scottish Government, 2012. SIMD: Background and Methodology.

[bib41] Scottish Government, 2014. Scottish Government Urban Rural Classification.

[bib42] Sedgwick P. (2015). Bias in observational study designs: cross sectional studies. BMJ.

[bib43] Smith O. (2016). Commute well-being differences by mode: evidence from Portland, Oregon, USA. J. Transp. Health.

[bib44] St-Louis E., Manaugh K., van Lierop D., El-Geneidy A. (2014). The happy commuter: a comparison of commuter satisfaction across modes. Transp. Res. F – Traffic.

[bib45] Sustrans Scotland, 2011. Evidence to the I&CI Committee on the Draft Budget 2012–13 and Spending Review 2011 dinburgh, Scotland.

[bib46] Transport Scotland (2014). Transport and Travel in Scotland: Road Traffic.

[bib47] Wener R.E., Evans G.W. (2011). Comparing stress of car and train commuters. Transp. Res. F – Traffic.

[bib48] Whybrow P. (2014). Physical Activity and Urban Living: A Mixed Methods Analysis of How Urban Form Influences Walking in Scotland.

[bib49] World Health Organisation (2015). Global Action Plan for the Prevention and Control of Noncommunicable Diseases 2013–2020.

[bib50] Ye R., Titheridge H. (2016). Satisfaction with the commute: the role of travel mode choice, built environment and attitudes. Trans. Res. D – Transp. Environ..

