# Supplementary material for: Changes over time in population level transport satisfaction and mode of travel: A 13 year repeat cross-sectional study, UK
Source: J Transp Health. 2017 Sep;6:366–78. doi: 10.1016/j.jth.2017.03.012 (PMC5633015; doi:10.1016/j.jth.2017.03.012)
Supplement: Supplementary Table 2 — Supplementary material [file mmc2.docx]

***Supplementary Table 1: Likelihood of journey satifaction, individual level variables, and contextual level variables, by individual journey destination: THAW 1 (1997)***

****** Note: Binomial distribution, 2nd order linearisation and PQL estimation. OR: Odds ratio, LL: lower level, UL: upper level, CI: confidence level

***Supplementary Table 2: Likelihood of journey satifaction, individual level variables, and contextual level variables, by individual journey destination: THAW 2 (2010)***

 Note: Binomial distribution, 2nd order linearisation and PQL estimation. OR: Odds ratio, LL: lower level, UL: upper level, CI: confidence level
